# Study on the Protective Effect of Special Electromagnetic Field Treated Water and Far Infrared Rays on LPS-Induced ARDS Rats

**DOI:** 10.1155/2019/5902701

**Published:** 2019-06-09

**Authors:** Ruiyin Huang, Yan Li, Xin Li, Qian Wang, Changyong Luo, Xu Liang, Lijie Liu, Xin Wang, Yuande Wu

**Affiliations:** ^1^Dongzhimen Hospital, Beijing University of Chinese Medicine, No. 5, Haiyuncang, Dongcheng District, Beijing 100700, China; ^2^Hospital of Traditional Chinese Medicine Chaoyang District, Beijing, China; ^3^Beijing University of Chinese Medicine, China; ^4^Biological Spectrum Institute, Guangdong Junfeng BFS Technology Co., Ltd., China; ^5^Peking Union Medical College, China

## Abstract

**Objective:**

To explore the protective effect of special electromagnetic field treated water (SEW) and far infrared rays (IFR) on endotoxin (lipopolysaccharide, LPS) induced ARDS rats and the effect on inflammatory factors.

**Methods:**

40 healthy male SD rats were randomly divided into 4 groups with 10 in each group. Preventive experiment: Adaptive feeding was carried out for 1 week according to animal feeding standards. Rats in SEW group drank SEW daily. Samely, rats in SEW and FIR group also drank SEW daily. Besides, rats in SEW and FIR group were also given far infrared rays for 20min/d. Rats in model group drank distilled water daily. After 7 days, rats in each group were injected with LPS (2 mg/kg) via the tail vein for making models. Rats in blank control group were given distilled water for 7 days, without modeling. All rats in the 4 groups were put to death under anesthesia 16 hours after modeling. Lung tissue and abdominal aortic blood were taken from these rats.

**Results:**

Pathological observation of lung and lung tissue indicated that rats in model group showed great pathological difference from rats in blank group. Rats in intervention group showed more symptomatic improvement in relation to alveolar and pulmonary interstitial congestion, edema, and inflammatory cell infiltration than rats in model group. The level of inflammatory factors like IL-1*β* and IL-6 in serum of rats in model group increased compared to blank control group (p<0.05). Comparing SEW group and SEW and FIR group with model group, levels of IL-1*β* and IL-6 in serum of rats both decreased remarkably (IL-I*β*: P < 0.05; IL-6: P < 0.01) while there was no obvious difference between SEW group and SEW and FIR group (p>0.05). The lung coefficient (LI) in SEW and FIR group was significantly lower than that in model group (*P*<0.01), which was higher than that of blank control group (*P* < 0.05), while there was no obvious difference between model group and SEW group (*P*>0.05). Compared with blank control group, lung permeability index (LPI) in model group showed no obvious difference (P>0.05).

**Conclusion:**

Special electromagnetic field treated water and far infrared rays can alleviate lung tissue damage of endotoxin-induced ARDS rats, relieving symptoms of alveolar and pulmonary interstitial congestion, edema, and inflammatory cell infiltration. The protective effect of special electromagnetic field treated water and far infrared rays on endotoxin-induced acute respiratory distress syndrome may result from their role in reducing the levels of IL-1*β* and IL-6 in serum and the expression level of p65 protein in lung tissue, in addition to reliving inflammatory response, lung coefficient, and lungs edema.

## 1. Introduction

Acute respiratory distress syndrome (ARDS) is a common clinical critical illness with complicated pathogenesis and poor therapeutic effect, and patients with ARDS are highly exposed to multiple organ failure and even death. It is a worldwide public health problem with a very high mortality rate of more than 40% [1, 2]. Clinically, respiratory support therapy [3] together with lung recruitment is usually adopted to relieve respiratory distress and systemic hypoxia. Besides, drug therapies like glucocorticoids, nitric oxide inhalation, pulmonary surfactant, and symptomatic treatment are often adopted though there is currently no reliable evidence from evidence-based medicine [4] for these therapies.

Pathological features of ARDS include pulmonary edema and inflammation of lung tissue. Inflammatory factors like IL-1*β* and IL-6 play important role in occurrence of ARDS; p65 involved NF-*κ*B passage is closely related to occurrence of ARDS, while lung coefficient is usually used for evaluating the degree of lung edema. Endothelial injury of pulmonary artery is often indicated by the increased lung coefficient.

In this study, in order to explore the preventive effects of special electromagnetic field treated water and far infrared rays on endotoxin-induced ARDS rats, special electromagnetic field treated water and far infrared rays (both provided by Guangdong Junfeng BFS Technology Co., Ltd., hereinafter referred to as SEW and FIR) were used for intervention in the animal models of endotoxin-induced ARDS rats, after which their effects on lung pathology, histology, and inflammatory factors in serum were observed.

## 2. Materials and Methods

### 2.1. Experimental Animals

40 healthy male SD rats, 8-week-old, weighing 200±10 g, were provided by Beijing Weitong Lihua Experimental Animal Technology Co., Ltd., and license number to these rats was SCXK (Beijing) 2012-0001. The rats were kept in BSL-2 Laboratory in Experimental Animal Center of Dongzhimen Hospital, Beijing University of Chinese Medicine, under 22-24°C, and the humidity was 50%-70%. Raised separately in different cages, these rats were given common granule and had free access to water. The experiment started after 1 week of adaptive feeding. All experiments about these rats were approved by Experimental Animal Welfare and Ethics Committee of Dongzhimen Hospital, Beijing University of Chinese Medicine.

### 2.2. Experimental Instruments and Reagents

 HC·TP11B·10 medical table balance (Beijing Medical Balance Factory), SC-3612 low speed centrifuge (Anhui Zhongke Zhongjia Scientific Instrument Co., Ltd.); Junfeng BFS water treatment & healthcare device (Guangdong Junfeng BFS Technology Co., Ltd.); Junfeng BFS treatment & healthcare device (Guangdong Junfeng BFS Technology Co., Ltd.); BH2 biological microscope (Japan OLYMPUS company); spectrophotometer (Unico (Shanghai) Instrument Co., Ltd.); and Ultraviolet analyzer (Beijing Shangbai Biology Company).

Endotoxin LPS (*Escherichia coli*, Sigma, USA, batch number L2880, 100mg), IL-1*β* ELISA kit (batch number A102453), and IL-6 ELISA kit (batch number A106893) were purchased from Aibokang (Shanghai) Trade Co., Ltd.; lung tissue pathology HE staining section was made by Pathology Department, Dongzhimen Hospital, Beijing University of Chinese Medicine.

### 2.3. Animal Grouping and Modeling

The 40 rats were divided into 4 groups (n=10) according to the random number table, namely, blank control group, model group, SEW group, and SEW and FIR (special electromagnetic field treated water and far infrared rays) group. Rats in each group were, respectively, weighed and recorded.

Intragastric administration was started 1 week after adaptive feeding. Rats in SEW group drank SEW daily. Samely, rats in SEW and FIR group also drank SEW daily. Besides, rats in SEW and FIR group were also given far infrared rays for 20min/d. Both the blank group and the model group were given distilled water for 7 consecutive days. On the 7th day, 6 hours after intragastric administration, the rats were not fasted. Rats in SEW group, SEW and FIR group, and model group were injected with endotoxin (LPS) solution (2 mg/kg body weight) while rats in blank control group were not given LPS. After LPS, all groups were put to death under anesthesia after 16 hours.

### 2.4. Sample Collection

#### 2.4.1. Blood Sample Collection and Treatment

10% chloral hydrate solution (3ml/kg) was intraperitoneally injected into the anesthetized rats. After that, we opened abdominal cavity of the rats, and then saw the abdominal aorta, took 4ml of abdominal aortic blood, and immediately injected it into heparinized test tube along the wall. After centrifugation at 3000 r·min-1 for 20 minutes, we took out the serum and stored it in a -80°C refrigerator.

#### 2.4.2. Collection of Pathological Specimens of Lung Tissue and HE Histological Staining

We fully exposed the thoracic cavity, removed the whole lung, and separated the right lower lung. After that, we fixed it with 10% paraformaldehyde, embedded it into paraffin, and then treated it with slice and HE stain to make HE staining pathological section. Lung histopathological HE staining sections were made by the Pathology Department, Dongzhimen Hospital, Beijing University of Chinese Medicine.

### 2.5. Observation Indicators and Methods

#### 2.5.1. Pathology of the Lungs

We fully exposed the chest cavity, removed the left lung intactly, and then fixed it with 10% paraformaldehyde. We observed with naked eyes pathological changes of the lungs, including the degree of blood stasis, the degree of swelling, and the color of the capsule.

#### 2.5.2. Lung Histopathology

We fully exposed the thoracic cavity, removed the whole lung, and separated the right lower lung. After that, we fixed it with 10% paraformaldehyde, embedded it into paraffin, and then treated it with slice and HE stain to make HE staining pathological section. The right middle lung was separated and stored in liquid nitrogen. Lung histopathological HE staining sections were made by the Pathology Department, Dongzhimen Hospital, Beijing University of Chinese Medicine. Histomorphological changes such as alveolar septal change, inflammatory cell infiltration, and pulmonary capillary congestion and edema were observed under optical microscope.

#### 2.5.3. Enzyme-Linked Immunosorbent Assay (ELISA)

To detect IL-1*β*, IL-6 levels in serum, 10% chloral hydrate solution (3 mL/kg) was intraperitoneally injected into anesthetized rats. After that, we opened the abdominal cavity, exposed the abdominal aorta, took 4 mL of abdomen aortic blood, and then injected it immediately into the heparinized test tube along the wall. After centrifugation at 3000 rpm for 20 minutes, we took out serum from the blood and then stored it in a -80°C refrigerator. The coated microbodies, which were previously coated with IL-1*β*, IL-6 antibodies, were sequentially added with specimens, standards, and HRP-labeled detection antibodies, and then they were incubated and thoroughly washed. The substrate was developed with TMB, and the absorbance (OD) was measured at 450 nm using a microplate reader to calculate the sample concentration.

#### 2.5.4. Western Blot

To detect p65 levels in lung tissue, according to instructions of the kit, quantitative gray analysis was performed by using Quantity One software to calculate the relative expression level of p65.

#### 2.5.5. Lung Coefficient (LI)

We took the whole lung and the whole trachea of the rats, cut the trachea between the 5 and 6 cartilage rings above the tracheal bifurcation, used the clean filter paper to absorb the blood and tissue fluid on the lung surface, and then weighed that with high precision balance. Pulmonary coefficient calculation method is as follows: lung coefficient (LI) = total lung wet weight (g) ÷ body weight (kg).

#### 2.5.6. Lung Permeability Index (LPI)

5 ml of blood was taken from rates through the inferior vena cava for separating plasma. After that, the rats were put into death immediately. Then, we opened thoracic cavity to expose the trachea, cut the trachea transversely from the upper end, and injected the upper end of the trachea for intragastric administration. The injection needle was fixed with a surgical line, and at the same time the right main bronchus was also ligated. The left lung was lavaged with 3 ml of normal saline. Bronchoalveolar lavage fluid (BALF) was collected after lavage. The lavage was performed 3 times in the same manner, after which lavage fluid was collected and the recovery rate was controlled at 80-90%. The alveolar lavage fluid was filtered through a double gauze and centrifuged at a high speed (3000 r/min) for 10 minutes, and then supernatant was taken. The protein content in alveolar lavage fluid and plasma was determined by ultraviolet absorption method. The calculation method of lung permeability index is as follows: lung permeability index (LPI) = BALF protein ÷ plasma protein.

### 2.6. Statistical Methods

Measurement data were expressed as average ± standard deviation ($\bar{x}\pm s$), and data processing was performed with SPSS 20.0 statistical software package. Variance analysis was used to compare the multiple groups. LSD was used to compare each two groups. The difference was statistically significant if p<0.05.

## 3. Results

### 3.1. General Conditions

The rats in the blank control group had good mental state, agile activity, rapid response, and shiny and supple hair. In the model group, the rats had poor mental state, less activity, slower response, darker coat, dull yellow, poor gloss, cleft lip and purpura, and excretion of thin stools. Rats in the treatment group had similar performance to the model group, but were not as severe as rats in model group.

### 3.2. Gross Observation of Lung Tissue

**Figure 1:**
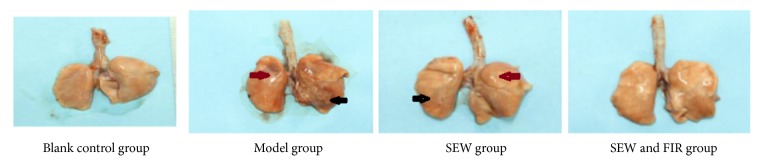
Effect of SEW, FIR on general observation of lung tissue in each group. Black arrow stands for ecchymosis while red arrow stands for edema.

The lung tissue of rats in blank control group showed no obvious abnormal changes, no volume increase, no edema, no ruddy color, and no blood stasis; the lung tissue of rats in model group showed increasement in volume, edema, and dark red spotted lesions of various sizes on the surface; the lung tissue of rats in the SEW group showed increasement in volume, edema, dark red color, and a small amount of scattered dark spots on the surface. The lung tissue of rats in SEW and FIR group was similar to the lung tissue in the blank group (see Figure 1).

### 3.3. Results of Pathological Observation of the Lung

**Figure 2:**
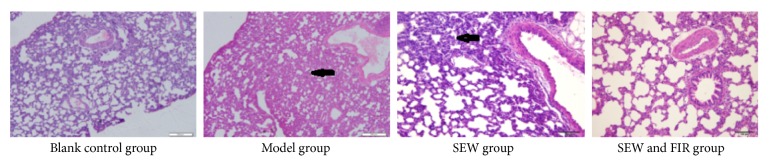
Effect of SEW, FIR on microphotographs H&E 20× of lung in each group. Black arrow stands for thickening of alveolar wall and shrink of alveolar space.

The lung tissue of rats in the blank control group showed clear and complete alveolar structure. The pathology of rats in the model group showed that the alveolar blunt was not clear, the structure was broken, the pulmonary interstitial edema appeared, the alveolar wall thickened, the alveolar space was reduced, a large amount of inflammatory exudate and red blood cells could be seen inside the alveolar space, a large amount of secretions could be seen in the bronchial cavity, the capillary wall congestion, and edema; compared with the model group, symptoms of rats in the SEW group together with the SEW and FIR group were improved, and the degree of improvement was similar (see Figure 2).

### 3.4. Comparison of IL-1*β* and IL-6 Levels in Serum

**Figure 3:**
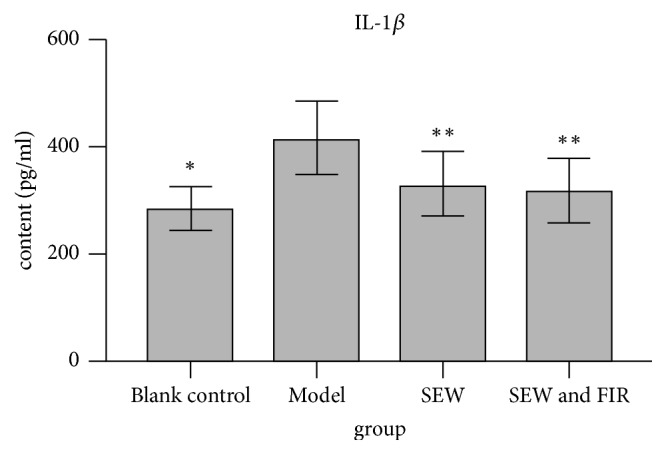
Effect of SEW, FIR on IL-1*β* of rats induced by LPS ($\overset{-}{x}\pm s$, n=10) (*∗*p<0.05 when compared to the model group, *∗∗* p< 0.01 when compared to the model group).

**Figure 4:**
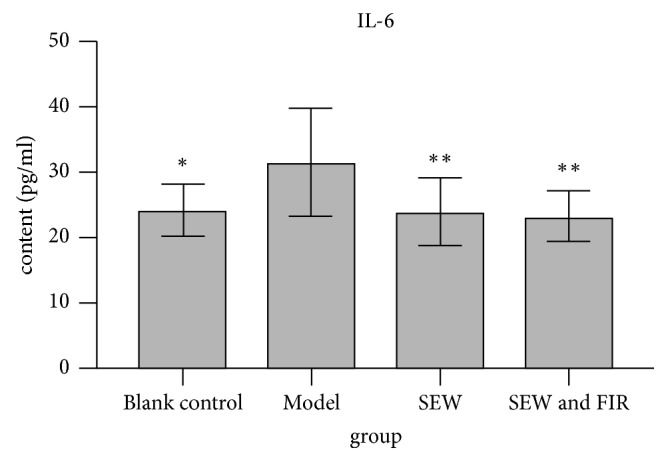
Effect of SEW, FIR on IL-6 of rats induced by LPS ($\overset{-}{x}\pm s$, n=10) (*∗*p<0.05 when compared to the model group, *∗∗* p< 0.01 when compared to the model group).

Compared with the blank control group, IL-1*β* and IL-6 levels in serum of rats in model group were both increased (p<0.05). Compared with the model group, the levels of IL-1*β* in the serum of the rats in the SEW group together with SEW and FIR group were significantly lower (p<0.01), and the levels of IL-6 were also significantly lower (p<0.01). There was no significant difference in levels of IL-1*β* and IL-6 between SEW group and SEW and FIR group (p>0.05). See Table 1, Figures 3 and 4.

### 3.5. Comparison of p65 Levels in Lung Tissue

**Figure 5:**
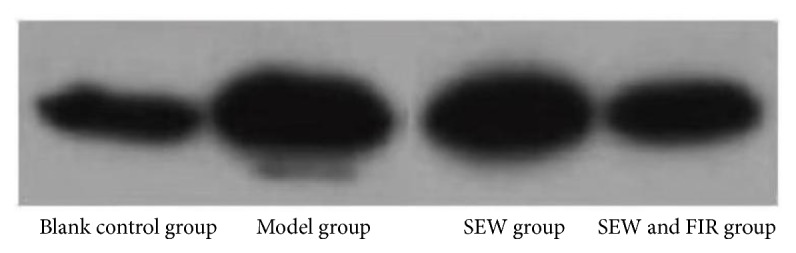
Effect of SEW, FIR on p65 of rats induced by LPS.

Compared with the blank control group, the model group had an elevated p65 (*P* < 0.05). Compared with the model group, p65 level was significantly lower in the SEW and FIR group (*P*<0.05), but there was no significant difference between the model group and SEW group (*P*>0.05). See Table 2 and Figure 5.

### 3.6. Lung Coefficient and Lung Permeability Index

The lung coefficient (LI) of rats in the model group was increased compared with the blank control group (*P*<0.05). Compared with the model group, the LI in the SEW and FIR group was significantly lower (*P*<0.01), but there was no significant difference between the model group and the SEW group (*P*>0.05). There was no significant difference in lung permeability index (LPI) between the model group and blank control group (*P>*0.05). Compared with the model group, the LPI in the SEW and FIR group was significantly lower (*P*<0.05). See Table 3.

## 4. Discussion

### 4.1. Acute Inflammatory Response Is a Key Link in the Pathogenesis and Development of ARDS

A study found that inflammatory response is closely related to acute respiratory distress syndrome [5], while inflammatory reactions, especially acute inflammatory reactions, play an important role in the early stages of acute respiratory distress syndrome. After being damaged by the injury, the body releases a large number of inflammatory factors, which can activate effector cells like macrophages and neutrophils. Under the stimulation of effector cells, the alveolar type II epithelial cells are damaged, and then swelling and necrosis occur, while apoptosis of neutral granulocyte is delayed, leading to tissue damage, excessive apoptosis of alveolar type II epithelial cells, [6], impaired sodium pathway function, alveolar fluid clearance disorder, and pulmonary edema. When alveolar edema is very severe, the entire alveolar can be filled, which leads to alveoli loss of alveolar function, alveolar collapse, and atelectasis; on the other hand, inflammatory factors stimulate the blood vessels, resulting in vascular endothelial damage, increased vascular permeability, increased pulmonary vascular exudation, a large amount of protein and edema fluid exudation, and aggravating pulmonary edema.

Changes in various plasma proteins and inflammatory cytokine levels during acute inflammatory reactions, such as the increased expression level of proinflammatory factors like tumor necrosis factor alpha, interleukin-1*β*, and interleukin-6 [7–9], initiated on the one hand the inflammatory cascade, activated nuclear transcription factors, and induced apoptosis; on the other hand, they increased the expression of inflammatory factors, and promoted damage repairmen IL-1*β* expression has relatively high level during the early stage of ARDS, which promotes the infiltration of monocytes and neutrophilic granulocytes to local inflammatory area. Then, monocytes and neutrophilic granulocytes release lysosomal enzyme and other cytokines. This improves the synthesis process of collagenases, stimulates the formation and release of a lot of inflammatory factors, and further leads to cell damage and worsens injury of lungs. Macrophages are stimulated by TNF-*α* and IL-1*β*, thus secreting IL-6, which upregulates the expression of adhesion molecules and other cytokines, and then trigger ARDS [10]. As a nuclear factor with multidirectional transcriptional regulation, NF-*κ*B is widely involved in physiological and pathological processes such as immunity, inflammation, and oxidative stress and is an important regulatory response in ARDS. Inflammation triggers the excessive activation of NF-*κ*B [11]. Inflammatory factors (such as IL-1*β*, IL-6) and NF-*κ*B can activate each other [12], which leads to the activation of NF-*κ*B and further stimulates the body to release a large number of inflammation-related substances. Then inflammation cascade reaction occurs, aggravating the lungs damage. The classical NF-*κ*B is a p65/p50 dimer structure. p50 contains a site that binds to DNA while p65 is mainly involved in the initiation of gene transcription and is also the main active form of NF-*κ*B. LPS activates NF- *κ*B to induce the release of a large number of inflammatory factors, which is the central link of ARDS. NF-*κ*B is the terminal control point of inflammatory response [13]. Inhibiting the activation of NF-*κ*B signaling pathway and reducing the activity of NF-*κ*B are beneficial to reduce lung inflammation, edema, and necrosis of alveolar cells and then further reduce the degree of lung injury. Therefore, blocking the activation of NF-*κ*B signaling pathway is likely to be an effective treatment for ARDS [14]. In respect of lung coefficient reflecting pulmonary edema in rats, there was a significant change in the SEW and FIR group; in respect of alveolar permeability index, the SEW and FIR group was significantly improved compared with the model group, suggesting that the SEW combined with FIR may have some preventive effect on ARDS.

### 4.2. Influence of SEW on ARDS

According to the safety evaluation experiment, SEW is safe and nontoxic and can improve the body immunity [15]; to some extent, it can also reduce red blood cells and platelet aggregation, reduce the degree of vascular endothelial edema, and improve vascular endothelial damage, bleeding, and other microcirculatory disorders [16]. The pathological process of ARDS is mainly about the damage of microvascular endothelial lung cells and alveolar epithelial cells [17]. Therefore, we believe that the partial protection of vascular endothelial cells by SEW may be one of the important mechanisms to improve the course of ARDS. Experiments have shown that SEW can reduce the inflammatory response in ARDS rats, which may be related to the reduction of interleukins.

### 4.3. Summary

The results of this experiment showed that ARDS rats had lung tissue damage, destroyed structure of lung tissue, pulmonary interstitial edema, inflammatory exudate in the alveoli, high level of IL-1*β*, IL-6 in serum and highly expressed p65 protein in lung tissues. Special electromagnetic field treated water combined with far infrared rays can improve the pathological damage of lung tissue of the rats and significantly reduce IL-1*β*, IL-6 in serum and the expression level of p65 protein in lung tissue. At the same time, it has the effect of reducing inflammation, significantly reducing lung coefficient, and reducing pulmonary edema, which shows that special electromagnetic field treated water combined with far infrared rays may be one of the effective ways to prevent ARDS.

In this experiment, the levels of inflammatory factors and p65 of rats in the model group were both increased, and after intervention by the special electromagnetic field treated water combined with far infrared rays, p65 level in lung tissue decreased, but there was no significant decrease of p65 level in rats in the SEW group. The serum levels of IL-1*β* and IL-6 decreased in both groups. Provided that that special electromagnetic field treated water and far infrared rays can inhibit inflammatory factors, there are three possibilities if we combine them together: first, by inhibiting the production of inflammatory factors, the activation of NF-*κ*B pathway is reduced, thereby reducing the inflammatory response; second, by inhibiting the activation of NF-*κ*B pathway, the release of inflammatory factors is reduced; third, special electromagnetic field treated water combined with far infrared rays could reduce the inflammatory response; whether it also blocks the activation of NF-*κ*B pathway while inhibiting the production of inflammatory factors needs further research in further experiments.

## Figures and Tables

**Table 1:** Effect of SEW, FIR on IL-1*β*, IL-6 of rats induced by LPS ($\overset{-}{x}\pm s$, n=10) (*∗*p<0.05 vs model group, *∗∗* p< 0.01 vs model group).

Group	IL-1*β* (pg/ml)	IL-6(pg/ml)
Blank control group	285.71±42.13*∗*	24.28±4.08*∗*
Model group	417.48±68.88	31.58±8.26
SEW group	330.97±59.25*∗∗*	24.02±5.27*∗∗*
SEW and FIR group	319.48±58.78*∗∗*	23.28±3.84*∗∗*

**Table 2:** Effect of SEW, FIR on P65 of rats induced by LPS ($\overset{-}{x}\pm s$, n=3) (*∗*p<0.05 vs model group, *∗∗* p< 0.01 vs model group).

Group	P65 (pg/ml)
Blank control group	21.94±1.79*∗*
Model group	35.89±5.95
SEW group	34.35±4.76
SEW and FIR group	23.55±2.26*∗∗*

**Table 3:** Effect of SEW, FIR on lung coefficient (LI) and lung permeability index (LPI) of rats induced by LPS ($\overset{-}{x}\pm s$, n=6) (*∗*p<0.05 vs model group, *∗∗* p< 0.01 vs model group).

Group	LI	LPI (×10^−2^)
Blank control group	4.93±0.17*∗∗*	0.33±0.06
Model group	7.16±0.37	0.46±0.11
SEW group	6.42±0.25	0.53±0.20
SEW and FIR group	6.11±0.40*∗∗*	0.24±0.04*∗*

## Data Availability

The data used to support the findings of this study are available from the corresponding author upon request.

## References

[1] Thompson B. T., Chambers R. C., Liu K. D. (2017). Acute respiratory distress syndrome. *The New England Journal of Medicine*.

[2] Phung T. T. B., Suzuki T., Phan P. H. (2017). Pathogen screening and prognostic factors in children with severe ARDS of pulmonary origin. *Pediatric Pulmonology*.

[3] Cavalcanti A. B., Suzumura É. A., Laranjeira L. N. (2017). Effect of lung recruitment and titrated positive end-expiratory pressure (PEEP) vs low PEEP on mortality in patients with acute respiratory distress syndrome: a randomized clinical trial. *Journal of the American Medical Association*.

[4] Emanuele R., Roberto F., Giacomo B. (2017). Definition and epidemiology of acute respiratory distress syndrome. *Annals of Translational Medicine*.

[5] Crimi E., Slutsky A. S. (2004). Inflammation and the acute respiratory distress syndrome. *Best Practice & Research Clinical Anaesthesiology*.

[6] Perl M., Chung C., Perl U. (2007). Fas-induced pulmonary apoptosis and inflammation during indirect acute lung injury. *American Journal of Respiratory and Critical Care Medicine*.

[7] Tanaka T., Narazaki M., Kishimoto T. (2014). Il-6 in inflammation, immunity, and disease. *Cold Spring Harbor Perspectives in Biology*.

[8] Liang J., Zhou Q., Zhang T. (2017). Changes and role evaluation of TNF-*α* and IL-1*β* in lung tissues of ARDS mice. *Chinese Journal of Cellular and Molecular Immunology*.

[9] Kalliolias G. D., Ivashkiv L. B. (2016). TNF biology, pathogenic mechanisms and emerging therapeutic strategies. *Nature Reviews Rheumatology*.

[10] Park W. Y., Goodman R. B. (2001). Cytokine balance in lungs of patients with acute respiratory distress syndrome. *American Journal of Respiratory and Critical Care Medicine*.

[11] Schwartz M. D., Moore E. E., Moore F. A. (1996). Nuclear factor-*κ*B is activated in alveolar macrophages from patients with acute respiratory distress syndrome. *Critical Care Medicine*.

[12] Steinberg J., Halter J., Schiller H. (2005). Chemically modified tetracycline prevents the development of septic shock and acute respiratory distress syndrome in a clinically applicable porcine model. *Shock*.

[13] Lu J., Chen X., Zhang Y. (2013). Astragalus polysaccharide induces anti-inflammatory effects dependent on AMPK activity in palmitatetreated RAW264.7 cells. *International Journal of Molecular Medicine*.

[14] Li W., Qiu X., Jiang H., Zhi Y., Fu J., Liu J. (2015). Ulinastatin inhibits the inflammation of LPS-induced acute lung injury in mice via regulation of AMPK/NF-*κ*B pathway. *International Immunopharmacology*.

[15] Liang X., Yuan L., Shi T. (2002). Evaluation on security and health care of water of frequency spectrum. *China Public Health*.

[16] Liu Y., Zhao X., Liu F. (2002). Effects of frequency spectrum water on leukocyte adhesion and microcirculation in rats. *Chinese Journal of Practical Medicine*.

[17] Umbrello M., Formenti P., Bolgiaghi L., Chiumello D. (2017). Current concepts of ARDS: A narrative review. *International Journal of Molecular Sciences*.

